# Supplementary material for: Unveiling the Bioactive Potential of the Invasive Jellyfish Phyllorhiza punctata Through Integrative Transcriptomic and Proteomic Analyses
Source: Biomolecules. 2025 Aug 4;15(8):1121. doi: 10.3390/biom15081121 (PMC12383608; doi:10.3390/biom15081121)
Supplement: Supplementary file 1 [file biomolecules-15-01121-s001.zip › Suplementary Material/FileS3/PEPFOLD4/XP_065063810_8490/clusters.html]

 PEP-FOLD clusters


Download archive of all models  here   

|  |  |  |
| --- | --- | --- |
| # | file | sOPEP |
| # cluster 1 - Size: 38 |  |  |
|  | XP\_065063810\_8490-model1 | -26.3321 |
|  | XP\_065063810\_8490-model1.1 | -25.8811 |
|  | XP\_065063810\_8490-model1.2 | -25.7094 |
|  | XP\_065063810\_8490-model1.3 | -25.6758 |
|  | XP\_065063810\_8490-model1.4 | -25.6247 |
|  | XP\_065063810\_8490-model1.5 | -25.6033 |
|  | XP\_065063810\_8490-model1.6 | -25.584 |
|  | XP\_065063810\_8490-model1.7 | -25.4943 |
|  | XP\_065063810\_8490-model1.8 | -25.4751 |
|  | XP\_065063810\_8490-model1.9 | -25.4408 |
|  | XP\_065063810\_8490-model1.10 | -25.3347 |
|  | XP\_065063810\_8490-model1.11 | -25.3101 |
|  | XP\_065063810\_8490-model1.12 | -25.2844 |
|  | XP\_065063810\_8490-model1.13 | -25.2385 |
|  | XP\_065063810\_8490-model1.14 | -25.203 |
|  | XP\_065063810\_8490-model1.15 | -25.1794 |
|  | XP\_065063810\_8490-model1.16 | -25.1752 |
|  | XP\_065063810\_8490-model1.17 | -25.1663 |
|  | XP\_065063810\_8490-model1.18 | -25.1276 |
|  | XP\_065063810\_8490-model1.19 | -25.0538 |
|  | XP\_065063810\_8490-model1.20 | -25.0143 |
|  | XP\_065063810\_8490-model1.21 | -25.0087 |
|  | XP\_065063810\_8490-model1.22 | -24.9769 |
|  | XP\_065063810\_8490-model1.23 | -24.9592 |
|  | XP\_065063810\_8490-model1.24 | -24.9249 |
|  | XP\_065063810\_8490-model1.25 | -24.9109 |
|  | XP\_065063810\_8490-model1.26 | -24.7915 |
|  | XP\_065063810\_8490-model1.27 | -24.7839 |
|  | XP\_065063810\_8490-model1.28 | -24.7412 |
|  | XP\_065063810\_8490-model1.29 | -24.5949 |
|  | XP\_065063810\_8490-model1.30 | -24.5571 |
|  | XP\_065063810\_8490-model1.31 | -24.5116 |
|  | XP\_065063810\_8490-model1.32 | -24.2529 |
|  | XP\_065063810\_8490-model1.33 | -24.2486 |
|  | XP\_065063810\_8490-model1.34 | -24.1405 |
|  | XP\_065063810\_8490-model1.35 | -23.5493 |
|  | XP\_065063810\_8490-model1.36 | -22.7997 |
|  | XP\_065063810\_8490-model1.37 | -22.6894 |
| # cluster 2 - Size: 35 |  |  |
|  | XP\_065063810\_8490-model2 | -26.2652 |
|  | XP\_065063810\_8490-model2.1 | -26.1601 |
|  | XP\_065063810\_8490-model2.2 | -26.1538 |
|  | XP\_065063810\_8490-model2.3 | -26.1078 |
|  | XP\_065063810\_8490-model2.4 | -25.8812 |
|  | XP\_065063810\_8490-model2.5 | -25.7416 |
|  | XP\_065063810\_8490-model2.6 | -25.71 |
|  | XP\_065063810\_8490-model2.7 | -25.666 |
|  | XP\_065063810\_8490-model2.8 | -25.6584 |
|  | XP\_065063810\_8490-model2.9 | -25.6462 |
|  | XP\_065063810\_8490-model2.10 | -25.4802 |
|  | XP\_065063810\_8490-model2.11 | -25.3585 |
|  | XP\_065063810\_8490-model2.12 | -25.3578 |
|  | XP\_065063810\_8490-model2.13 | -25.3432 |
|  | XP\_065063810\_8490-model2.14 | -25.3088 |
|  | XP\_065063810\_8490-model2.15 | -25.2764 |
|  | XP\_065063810\_8490-model2.16 | -25.2311 |
|  | XP\_065063810\_8490-model2.17 | -25.2244 |
|  | XP\_065063810\_8490-model2.18 | -25.2208 |
|  | XP\_065063810\_8490-model2.19 | -25.1812 |
|  | XP\_065063810\_8490-model2.20 | -25.1721 |
|  | XP\_065063810\_8490-model2.21 | -25.0845 |
|  | XP\_065063810\_8490-model2.22 | -25.0164 |
|  | XP\_065063810\_8490-model2.23 | -24.9938 |
|  | XP\_065063810\_8490-model2.24 | -24.9898 |
|  | XP\_065063810\_8490-model2.25 | -24.9746 |
|  | XP\_065063810\_8490-model2.26 | -24.9572 |
|  | XP\_065063810\_8490-model2.27 | -24.8631 |
|  | XP\_065063810\_8490-model2.28 | -24.8157 |
|  | XP\_065063810\_8490-model2.29 | -24.781 |
|  | XP\_065063810\_8490-model2.30 | -24.6052 |
|  | XP\_065063810\_8490-model2.31 | -24.5967 |
|  | XP\_065063810\_8490-model2.32 | -24.4326 |
|  | XP\_065063810\_8490-model2.33 | -24.391 |
|  | XP\_065063810\_8490-model2.34 | -23.0454 |
| # cluster 3 - Size: 3 |  |  |
|  | XP\_065063810\_8490-model3 | -26.2542 |
|  | XP\_065063810\_8490-model3.1 | -25.9345 |
|  | XP\_065063810\_8490-model3.2 | -25.8352 |
| # cluster 4 - Size: 11 |  |  |
|  | XP\_065063810\_8490-model4 | -26.1838 |
|  | XP\_065063810\_8490-model4.1 | -25.9055 |
|  | XP\_065063810\_8490-model4.2 | -25.7977 |
|  | XP\_065063810\_8490-model4.3 | -25.7668 |
|  | XP\_065063810\_8490-model4.4 | -25.5357 |
|  | XP\_065063810\_8490-model4.5 | -25.3227 |
|  | XP\_065063810\_8490-model4.6 | -25.2377 |
|  | XP\_065063810\_8490-model4.7 | -25.2016 |
|  | XP\_065063810\_8490-model4.8 | -24.2787 |
|  | XP\_065063810\_8490-model4.9 | -23.7395 |
|  | XP\_065063810\_8490-model4.10 | -22.5284 |
| # cluster 5 - Size: 1 |  |  |
|  | XP\_065063810\_8490-model5 | -25.792 |
| # cluster 6 - Size: 2 |  |  |
|  | XP\_065063810\_8490-model6 | -25.6243 |
|  | XP\_065063810\_8490-model6.1 | -25.0036 |
| # cluster 7 - Size: 1 |  |  |
|  | XP\_065063810\_8490-model7 | -24.7289 |
| # cluster 8 - Size: 3 |  |  |
|  | XP\_065063810\_8490-model8 | -24.6763 |
|  | XP\_065063810\_8490-model8.1 | -23.9893 |
|  | XP\_065063810\_8490-model8.2 | -23.664 |
| # cluster 9 - Size: 1 |  |  |
|  | XP\_065063810\_8490-model9 | -24.3725 |
| # cluster 10 - Size: 1 |  |  |
|  | XP\_065063810\_8490-model10 | -23.9634 |
| # cluster 11 - Size: 1 |  |  |
|  | XP\_065063810\_8490-model11 | -20.078 |
| # cluster 12 - Size: 3 |  |  |
|  | XP\_065063810\_8490-model12 | -19.6112 |
|  | XP\_065063810\_8490-model12.1 | -18.5957 |
|  | XP\_065063810\_8490-model12.2 | -18.5838 |
|  |  |  |
